# Facilitators and Barriers to Implementation of a Childhood Tuberculosis Control Program in Bangladesh: A Mixed-Methods Study from BRAC Urban DOTS Centres in Dhaka

**DOI:** 10.3390/nursrep12020036

**Published:** 2022-05-10

**Authors:** Sandesh Pantha, Ma. Jennylyn Aguinaldo, S. M. Hasan-ul-Bari, Sayantan Chowdhury, Ugyen Dendup, Rajat Das Gupta, Ipsita Sutradhar, Rahamatul Bari, Malabika Sarker

**Affiliations:** 1 James P Grant School of Public Health, Dhaka 1212, Bangladesh; jennylyn.aguinaldo@gmail.com (M.J.A.); sm.hasanulbari@eleve.ehesp.fr (S.M.H.-u.-B.); passionofsayan@gmail.com (S.C.); udendup@health.gov.bt (U.D.); rajatdas@email.sc.edu (R.D.G.); ipsita.sutradhar@bracu.ac.bd (I.S.); rahmatul@bracu.ac.bd (R.B.); malabika@bracu.ac.bd (M.S.); 2 School of Nursing and Midwifery, La Trobe University, Melbourne 3086, Australia; 3 International Labour Organisation, Manila 1200, Philippines; 4 French School of Public Health, Leon-Bernard, 35043 Rennes, France; 5 United Nations Population Fund (UNFPA), Dhaka 1207, Bangladesh; 6 Ministry of Health, Kawajangsa, Thimphu 726, Bhutan; 7 Department of Epidemiology and Biostatistics, Arnold School of Public Health, University of South Carolina, Columbia, SC 29208, USA; 8 Heidelberg Institute of Global Health, Heidelberg University, Neuenheimer Feld, 69120 Heidelberg, Germany

**Keywords:** childhood TB, child TB, implementation, diagnosis, treatment, facilitators, barriers

## Abstract

The case detection rate of childhood tuberculosis in Bangladesh is 4%, far below the World Health Organization predicted rate of 10–15% for a country with a high burden of tuberculosis. A concurrent triangulation mixed-methods study was carried out in eight urban DOTS (Directly Observed Treatment, Short-course) centres to investigate the factors contributing to the diagnosis and treatment of childhood tuberculosis. Front-line health care workers (*Shasthya Shebika*) (*n* = 111) were surveyed to understand knowledge, attitude, and practice (KAP) of the diagnosis and treatment of childhood tuberculosis. In-depth interviews were conducted with field workers (*n* = 32) and mothers of TB cases (*n* = 4). Stakeholders involved in implementing the tuberculosis program (*n* = 9) participated in the key informant interviews. Knowledge of *Shasthya Shebika* was associated with the components addressed during refresher training (*p* = 0.02). Government stewardship, presence of specific guidelines, knowledge and capacity building of front-line health workers were identified as the key facilitators. Frequent turnover of key managerial positions in the government, stigma, delays in seeking care, lack of diagnostic facilities, and poor engagement of private practitioners were identified as major constraints. It was identified that the government should focus on improving diagnostic capacities, conduct research on childhood tuberculosis, and produce awareness materials.

## 1. Introduction

Tuberculosis is a bacterial infection caused by *Mycobacterium tuberculosis* [1]. It has been estimated that about 40 million people are infected with tuberculosis (TB) in 2022 of which 3.5 million are children [2]. Between 2016 and 2019, the annual death attributed to childhood TB was approximately 200,000 [3,4]. South Asia contributes to two in five cases and one in three TB deaths globally [5]. There is a huge gap between the estimated and reported number of TB cases identified and treated. For example, in 2020, less than 60% of the estimated TB cases were reported [6]. Less than half of the children with TB received treatment between 2018 and 2020 [7]. The World Health Organisation (WHO) estimates that in a country with a high burden of TB, children are likely to contribute about 10%–15% of total TB cases [1,8,9]. The number of reported cases of childhood TB has improved over the past few years but is much less than the projected figures [3,5]. In contrast, the number of childhood TB cases identified, reported, or treated in these high burden countries is far below the expected numbers [10]. For example, children below 15 years comprised less than five percent of the total cases reported in Bangladesh between 2015–2019 [11,12,13]. To reduce the global burden of tuberculosis, the World Health Organisation (WHO) introduced the ‘End TB strategy’ in 2015 with a vision of zero avoidable deaths, disease, and sufferings from TB by 2030 [14]. The ‘End TB strategy’ advocates for global partnership and has three strategic pillars—1. patient-centric care, 2. bold policies and supportive systems, and 3. intensified research and innovation [14]. Although tuberculosis diagnosis and treatment has gained wider attention because of this initiative, the number of childhood TB cases has not increased as estimated [3,5].

There could be potential challenges to improve diagnosis and treatment of childhood TB. Lack of adequate knowledge, attitude, and awareness (KAP) among front-line health care may be one of the important factors that delay identification of active cases. Bjerrum et al. (2012) interviewed 29 front-line health care workers in rural Tanzania to understand health workers’ perceptions of childhood TB [15]. The authors report that none of the health care workers interviewed in the study recognized childhood TB as an emerging problem. Besides, as sputum production is difficult in children, particularly below five years of age, relying on the conventional diagnostic criteria (positive sputum microscopy in a case with constitutional symptoms of tuberculosis—fever, cough >2 weeks, and weight loss) may lead to missed opportunities [16,17]. Furthermore, low sensitivity of the diagnostic criteria, financial constraints, and lack of modern laboratory tests (GeneXpert and X-rays) may contribute to delays in early diagnosis of suspected cases [9,12,17]. In addition, TB is often not suspected in a sick child due to the preconception that BCG (Bacillus Calmette–Guerin) vaccination protects children from tuberculosis [17,18]. The lack of child-friendly drugs [19,20], inadequate supply [17], and difficulty in administrating medicines [21] may contribute to poor treatment adherence in children. Therefore, to overcome these diagnostic and therapeutic difficulties, it is important to explore the existing implementation and find out what are the good attributes and what needs to be improved or modified.

Bangladesh, globally known for its successful implementation of an innovative tuberculosis control program, is struggling to identify children with TB [22]. Bangladesh is one of the ten countries with the highest gap between estimated and reported cases of TB [5]. The government of Bangladesh adapted the WHO (World Health Organisation) recommended guidelines for childhood TB [23,24] in 2012 [25]. However, even after five years of incorporating the specific guidelines, the childhood TB case numbers have been steady at below 5% [11,12,13]. Bangladesh has one of the highest treatment success rates for TB in the world [12]. However, the challenges specific to the treatment of childhood TB in Bangladesh has not been previously explored. The aim of this study is to explore cross-cutting issues in the strategies used for the implementation (implementation pathway) of diagnosis and treatment of a childhood TB control program in Bangladesh based on the three strategic pillars of the “End TB Strategy” [14].

## 2. Materials and Methods

### 2.1. Implementation of Childhood TB in Bangladesh

**Figure 1:**
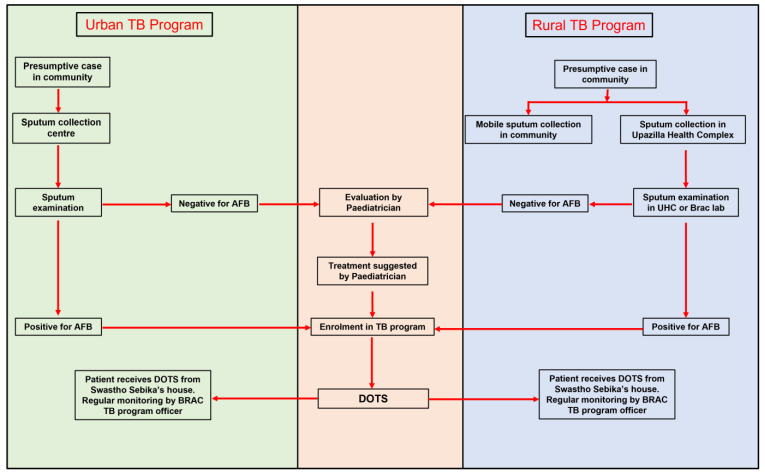
Implementation pathway of TB diagnosis and treatment in Bangladesh.

Figure 1 illustrates the implementation of TB diagnosis and treatment in Bangladesh. A presumptive child TB case is identified in the community by the frontline volunteer workers and sent for sputum collection. Sputum is collected either at mobile sputum collection camps or fixed TB clinics. These fixed TB clinics include DOTS (Directly Observed Treatment Short course) centres in Urban areas and UHC (Upazilla Health Complex) in the rural areas). Treatment (Four drug regimen for the first two months—Rifampicin, Isoinazid, Ethambutol, and Pyrazinamide and two-drug regimen—rifampicin and isoniazid—for the next four months) is initiated if sputum smear for the acid-fast bacillus (AFB) stain is positive for *Mycobacterium tuberculosis*. A case with a negative sputum examination is referred to a pediatrician. Treatment for such children is initiated upon recommendation from a pediatrician. Cases are registered at the DOTS centre and medication is handed over to a frontline worker who lives close to the case. Every morning, the person must visit a frontline worker’s house for medicine. If a frontline healthcare worker is not available to provide medicine, alternate provisions are made to offer treatment under direct observation of a community leader or a family member. Treatment is monitored fortnightly by the supervisors.

### 2.2. Study Design and Setting

We used a concurrent triangulation design with convergent model, a variant of the mixed-method approach. In a concurrent design, both quantitative and qualitative data are collected simultaneously, analysed separately, and results are triangulated with each other [26]. We used a mixed methods approach to enhance the strength of the study [26,27]. We collected data at two levels—policy level and the field level. Policy level data was obtained from the stakeholders involved in planning, implementation, monitoring, and evaluation of childhood TB program in Bangladesh. Field-level data, both qualitative and quantitative, were collected from eight urban DOTS centres under Dhaka City Corporation.

The DOTS centre in Dhaka are operated by BRAC, a non-for-profit organisation that implements the national TB control program in 42 out of 64 districts in Bangladesh [11,12]. There are 24 urban DOTS centres in Dhaka City Corporation, each with a catchment area population of about 100,000. We obtained the number of new cases of childhood TB for 18 months (January 2016–June 2017) from the monitoring and evaluation division of BRAC TB program. Eight centres (four each with the highest and lowest number of new cases) were selected as the study sites. These centres will be called “high performing centres” and “low performing centres” for the rest of the manuscript. The study was purposefully conducted in the high performing and low performing centres to identify if any factors influenced differences in case notification.

Table 1 shows detailed information of the study population, number of respondents, and the objectives addressed by different components of our study.

#### 2.2.1. Quantitative Component

We surveyed front-line healthcare workers (*Shasthya Shebika—(SS*)) to investigate the knowledge, attitude, and practices of diagnosis and treatment of childhood TB. The SS are front-line voluntary workers in BRAC program who provide door to door services in the community. They identify suspected cases based on their symptoms (cough for more than two weeks and fever), bring, or encourage presumptive cases to visit the DOTS centre for sputum examination, and provide treatment after diagnosis. They receive basic and periodic refresher training to upgrade their knowledge [28].

Eligibility for survey was based on two criteria—1. lives within a radius of 5 kilometres from DOTS centre and 2. Has not provided treatment to a child over 12 months prior to data collection. Eligible participants were identified through the program organizer of the respective DOTS centre. We purposefully included SS not providing TB medication to a child for the past 12 months to address one of the potential confounding factors as we anticipate that SS providing a treatment to child TB in the recent period will have better knowledge compared to others.

We did not find any data on the KAP scores on childhood TB among the frontline health workers in Bangladesh. We therefore determined the sample size for the survey based on an assumption that 50% of the SS have adequate knowledge of childhood TB. With 80% power, 10% margin of error, and 20% non-participation rate, the required sample size was 116. The required participant number was calculated proportionately based on the total number of SS working in these eight centres.

#### 2.2.2. Qualitative Component

In-Depth-Interviews (IDI) (*n* = 32) were conducted with the front-line health care workers (Table 1) and mothers of children with active tuberculosis to explore the field level implementation and consumer experiences of service uptake. To be eligible for the interview, health workers need to be working for at least six months before data collection. The SS included in the survey did not participate in the IDI.

We conducted the Key Informant Interview (KII) (*n* = 9) with the senior officials engaged in the implementation of the TB control program. Through the KII, we investigated implementation pathway, strategic components, and initiatives taken for case detection and management of childhood TB.

### 2.3. Study Tools

A structured questionnaire was developed for the survey following the guidelines recommended by WHO for developing KAP surveys [29]. We adopted the health workers KAP questionnaire on TB care used in Mozambique [30]. Questions specific to childhood TB was added with input from previous studies on TB in children [17,31,32]. The questionnaire was translated in Bangla. An English version of the questionnaire is available as a Supplementary Materials (Supplementary Material S1: Questionnaire KAP survey). The IDI and KII were conducted using interview guidelines. These guidelines were translated into Bangla.

Survey questionnaire (*n* = 10) and IDI guidelines (*n* = 3) were pre-tested in a Dhaka Urban DOTS centre which was not a site for study data collection. Feedback from the pre-test was incorporated in the final study tools.

### 2.4. Data Collection

Data collection was carried out in December 2017. Field level surveys and interviews were conducted in a private area inside the DOTS centre on the day of their monthly meeting. Bengali speaking team members (SP, SMH, and SC) approached the participants, explained the study objectives to them and obtained their informed written consent. The survey was administered by four trained data enumerators under the direct supervision of the researchers. The IDI’s were conducted in Bangla by the Bengali speaking team members. All field level participants were offered light snacks and thanked for their participation.

The key informants were interviewed in English by the members of the research team (JA, SC, SMH, SP, and UD). The KII was carried out as per participants’ convenient time and place.

### 2.5. Data Analysis

#### 2.5.1. Quantitative Data Analysis

Data were entered in Microsoft Excel. Two researchers independently entered the data. Any differences in data entry were verified with the survey form by a third researcher. Data was imported, cleaned, and analysed in Stata 13.0. Descriptive analysis was carried out using frequency and percentage. The number of questions that evaluated knowledge, attitude, and practice domains were 61, 12, and 16 respectively. Each correct and incorrect answer on knowledge, attitude, and practice were scored ‘1′ and ‘0′ respectively. The total scores on each domain of knowledge, attitude, and practices were calculated and categorized as “Poor”, “Average” and “Good” with a 33 percent and 67 percent (of maximum score) as cut-off value. A bivariate analysis was done to identify an association between the KAP scores and demographic characteristics of the respondents that included age, level of education, duration of work, duration of treating a child with tuberculosis, performance of DOTS centre and number of cases treated within past 12 months. Chi-square and Fisher Exact tests were used wherever applicable.

#### 2.5.2. Qualitative Data Analysis

The IDI and KII conducted in Bangla (one of the key informants mostly answered in Bangla) were translated into English, whereas those conducted in English were transcribed verbatim. Translation was done by the data enumerators and verified by the Bengali speaking members of research team (SP, SMH, and SC). Four A-priori codes (with sub-codes) were identified—1. facilitator-diagnosis, 2. facilitator-treatment, 3. barrier-diagnosis, and 4. barrier-treatment. Some of the sub-codes emerged during coding. For example, treatment bond money was identified during coding. Table 2 provides a description of the codes and sub-codes. Thematic data analysis was carried out by the researchers (SP, SS, SHM, JA, and UD) in coordination with and under supervision of the supervisors (RDG, PH, and MS). The transcripts were read repeatedly in order to be familiar with the interview scripts. Two researchers coded the first ten interviews to ensure consistency in coding. The rest of the interviews were coded by a single researcher. Coding was done using Atlas.ti 7. We clustered, compared, and categorized the data. Data display was prepared to identify patterns emerging from the data. Data validation was done by triangulation with the field and program level data and with the field observations and notes taken during data collection. We will present the facilitators and barriers based on the three strategic pillars of the ‘End TB strategy’.

### 2.6. Ethics

Ethical approval was obtained from the institutional review board at James P Grant school of public health (JPGSPH) (Approval Number: 2017-MPH-SLP16). Approval was obtained from the BRAC tuberculosis program. Participants were informed of research objectives and were assured of anonymity and confidentiality. A written informed consent was obtained from all participants. All interviews were audio recorded after obtaining verbal consent from the participants. De-identified data was used for analysis and preserved as per JPGSPH’s institutional policy.

## 3. Results

This study complies with the guidelines of the Standards for Reporting Implementation Studies (StaRI) checklist (Supplementary Material S2: StaRI Checklist) [33].

### 3.1. Survey Findings

The survey was completed by 111 SS (response rate = 95.6%). Table 3 shows demographic characteristics of the respondents. The mean (±standard deviation) age of SS respondents was 40.3 (±0.9) years and average duration of work was 9.5 (±0.3) years. About half (48.7%) of the respondents were from high performing centres. The average number of adult TB cases treated over the last 12 months was 8 (±0.7). The average duration of treating a child TB case was 6 years (±1.1). Majority (89%) recalled that refresher training addressed over five out of seven components of tuberculosis program—diagnosis, treatment, disease prevention, isoniazid prevention therapy (IPT), counselling, stigma, and field level supply chain of the medicine.

#### 3.1.1. Knowledge on Childhood TB

Most of the participants (94%) knew children can be infected with TB. Eight possible symptoms (persistent cough, weight loss, loss of appetite, lethargy, blood-stained sputum, weakness, neck mass, and mass in the back) were identified by almost 90% of the respondents. One in four (23%) didn’t know that fever of ≥2 weeks is one of the cardinal symptoms of tuberculosis in children. Less than a third had correct information about transmission during childbirth (25%) and through breastfeeding (30%). Only half (51%) identified that BCG vaccination could protect children from TB. About 96% knew TB is curable in children and 90% believed that adult drugs cannot be provided to them. Only one-third of the respondents knew that duration of treatment of TB in children is same as in adults.

#### 3.1.2. Attitude on Childhood TB

Almost all respondents identified that finding new cases and community engagement were essential for the control of TB infection. Two out of three respondents agreed that public awareness of childhood TB was not sufficient.

#### 3.1.3. Practice Regarding Childhood TB

Most of the respondents (>95%) counselled that TB was a curable disease, importance of compliance and on the duration of TB treatment. The majority of the respondents practiced the correct recommendations when providing medications and monitoring the drug therapy.

#### 3.1.4. Bivariate Analysis

Total score on knowledge, attitude and practice domains were 61, 12, and 16 respectively. The mean (±S.D.) for the total score on knowledge, attitude, and practices were 43.6 (± 0.5), 10.8 (±0.9) and 13.2 (±0.2), respectively. Mean score on knowledge of symptoms of childhood tuberculosis (*n* = 13) was 11.1 (95% CI 10.9–11.5); mean score on knowledge of risk factors (*n* = 5) was 4.3 (95% CI 4.2–4.5). However, mean knowledge on routes of transmission (*n* = 10) was 4.3 (95% CI 4.0–4.5). Almost all of them had good scores on different components of attitude and practice related to diagnosis and treatment of tuberculosis. Detailed information on the scores on different domains of knowledge, attitude, and practices is available as a Supplementary Materials (Supplementary Material S3: KAP Scores).

The knowledge of symptoms of childhood TB was significantly associated with the number of adult TB cases treated by SS in the preceding 12 months (*p* = 0.02) and components of TB care addressed during training (*p* = 0.04). There was no association of KAP scores on respondent characteristics (age, duration of work, duration of treating child cases, number of cases treated). There was no difference in KAP scores among high performing and low performing centres (Supplementary Material S3: KAP Scores).

### 3.2. Qualitative Findings

Table 4 presents the key facilitators and barriers to implementation of childhood TB for the three strategic pillars of “the End TB Strategy”.

#### 3.2.1. Pillar 1: Integrated Patient-Centred Care

##### Training and Capacity Building

The key informants reinforced that the front-line healthcare workers were adequately trained and played a key role in identifying potential cases and ensuring treatment compliance. A program manager at the national tuberculosis centre, which is the governing body for all activities for tuberculosis control in Bangladesh, informed us that the government of Bangladesh has developed training modules and guidelines for different healthcare professionals so that they can provide an adequate amount of information specific to the position. For example, periodic refresher training was provided to all SS. Each refresher training for SS addressed seven components (diagnosis, treatment, prevention, IPT, counselling, stigma, and supply chain) for diagnosis and treatment TB.

*“In the field level, their (community volunteers’) role is to identify the suspects; they need to learn these criteria (for diagnosis) … they need to know how to refer these cases and when diagnosed, how to link these cases to DOTS provider...”*

(KII 4)

The front-line healthcare workers reinforced the information obtained from the key informants that the periodic refresher training helped SS develop confidence on talking about preventive strategies, diagnosis criteria, and treatment for childhood TB.

##### The Complexity of Diagnosis of Childhood TB

It was reported that even doctors do not suspect TB during the initial workup of a sick child. Most of the times, children were diagnosed with TB only after physicians failed to reach other diagnosis.

*“When other diagnosis cannot prove, then the physician will be thinking that it might be a case of tuberculosis”*

(KII 1)

Key informants mentioned that diagnostic advances like GeneXpert contributed to the increased case number. However, it was further highlighted that investigations like GeneXpert, biopsy and culture were not available in most of the government health facilities (Upazilla health complex). Field organizers and SS emphasized that due to lack of proper investigations, people had to travel to the district hospital and other tertiary care hospitals resulting further financial constraints to poor people.

##### Guideline for Childhood TB

The guidelines for management of childhood TB in 2012 was identified as a key turning point in identifying a child with TB. This guideline eliminated need for a mandatory positive sputum examination to initiate the treatment and facilitated symptomatic diagnosis for under-5 children.

##### Financial Assistance

A program officer at national tuberculosis control program confirmed that sputum examination and Mantoux tests were provided free of cost from all the health facilities, either public or private. Monitoring and evaluation manager at BRAC TB program mentioned that financial support contributed to increased case detection.

*“…financial support is big term… We started this journey around 2012. If you see the case trend from 2013 there is big jump in 2013, a sharp jump (in the number of TB patients)”*

(KII 10).

It was revealed that sputum negative cases, referred from BRAC DOTS centre, were eligible to receive a financial support as reimbursement of expenses for diagnostic tests related to TB (maximum of 2000 Taka, equivalent to US$ 25 in 2017). But field staff reported that most children were often unable to use this facility as they cannot produce sputum.

##### Awareness of Childhood TB

Participants highlighted lack of availability of information, education, and communication (IEC) materials on childhood TB. During our field work, we observed that IEC materials on childhood TB was present only in the two DOTS centres. A key informant from the national tuberculosis program (NTP) informed that the government was working on audio-visual materials for childhood TB to be aired through national media. Key informants from the government and implementing partners argued that it was the responsibility of other partner to develop and distribute IEC materials for awareness.

##### Underreporting of TB Cases

The key informants highlighted that there is underreporting of the TB cases, mainly from the private practitioners and informal healthcare workers.

*“…now we have covered around 62% of the estimated cases under detection. So, we are thinking that those missing cases may be in private sectors. Either not diagnosed at all or if diagnosed may be in the private sectors…”*

(KII 6).

They further reinforced that, due to stigma, people do not want to go to the DOTS centre to take free medications, and instead may purchase medications from the pharmacies, which are not recorded. To overcome the challenge, the government-initiated training and capacity building of the private practitioners. The key informant at NTP said that the government has developed a mobile application for case notification and will reinforce its implementation.

*“So, then government circulated that gadget as mandatory notification. But only gadget cannot ensure. We have to make it functional (operationalize) this”*

(KII 6).

##### Delay in Diagnosis

**Figure 2:**
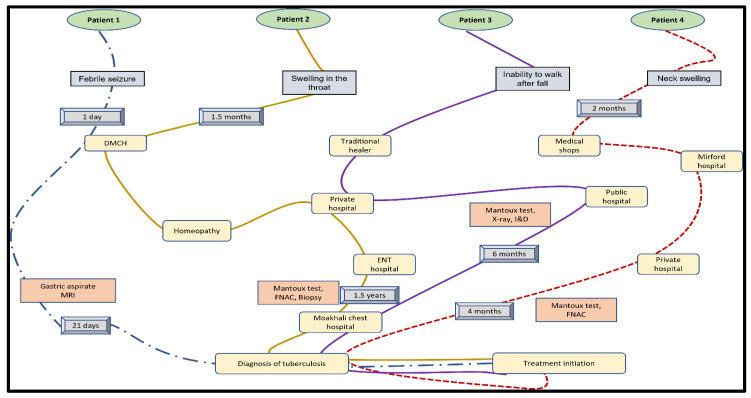
Pathway of diagnosis for four childhood TB cases.

The in-depth interview with parents of childhood TB cases revealed that three out of four cases had at least six months of delay in diagnosis (Figure 2). These children went back and forth between traditional healers, public, and private health facilities before visiting tertiary care hospitals. All mothers said that despite having a range of investigations—for example, fine needle aspiration cytology (FNAC), GeneXpert, and magnetic resonance imaging (MRI)—TB was suspected only when other conditions were ruled out.

##### Child Friendly Regimen

The government introduced child-friendly medication with fixed drug combinations (FDC) for children. An orally dispersible tablet was available with 2FDC (Isoniazid, Rifampicin) and 3FDC (Rifampicin, Ethambutol, and Pyrazinamide). However, there was no child-friendly regimen with 4FDC (Isoniazid, Rifampicin, Ethambutol, Pyrazinamide). Those who need to take 4FDC need to take 3FDC and isoniazid. Taking two tablets instead of one was not easy for children. Besides, children often vomit out medicine. Difficulty in administrating medicine was an important factor that hindered treatment in children. The IDI with mothers of two TB cases (one aged thirteen months and another aged 6 years) admitted that it was difficult to give medicine to small children.

*“Children don’t want to take it, they want to throw away. And different types of problem”*

(IDI 26).

##### Active Surveillance during Diagnosis and Treatment

Any presumptive cases sent for investigations were followed up until results were obtained.

*“To ensure they go for diagnosis, I go with them and help them with it, and if I can’t go, I contact them and ask whether they visited the doctor or not”*

(IDI 27).

The treatment of TB is provided under direct observation (DOTS) mostly by the SS. The BRAC program manager revealed strict supervision ensured higher compliance with treatment. In-depth-interviews with SS revealed that if patients do not come for medicine, they visited their (case’s) home to provide medicine and provide counselling on the importance of completing the total dose of medicine. It was acknowledged that making a child take medication for six months was a Herculean task.

*“The medicine is for 6 months. After completing the dose for 2 months, the child starts feeling better. That time child does not want to continue medicine. These are the barriers. That is why our duty (supervision) is going on”*

(IDI-3).

Program/field organizers routinely visited patients’ home, cross-checked medicine with SS or DOTS provider to check compliance with the treatment.

Further, during treatment, all family members are monitored for symptoms. Every child under 5 years of age in the family is provided with isoniazid prevention therapy (IPT) for six months.

#### 3.2.2. Pillar 2: Bold Policies and Supportive Systems

A key informant from the WHO country office claimed that there was smooth coordination between NTP and the partner organisations.

*“In Bangladesh the government and non-government collaboration is excellent… the whole TB program in Bangladesh is being implemented by NGOs where government stewards the program”*

(KII 3).

Periodic policy review and addressing gaps and challenges through the Joint Monitoring Mission (JMM), conducted every 3 years, was one of the key reasons behind successful implementation of the TB control program in Bangladesh. For example, the JMM had identified shortage of trained human resources as a key challenge for childhood tuberculosis which was responded by intensified training of pediatrician and other health care workers. The government has introduced childhood TB management in MBBS (Bachelor of Medicine and Bachelor of Surgery) curriculum following suggestions from the JMM. Medical students are oriented on existing protocols with a hope of creating uniformity and reducing load to train one of the important groups of health professionals.

#### 3.2.3. Pillar 3: Intensified Research and Innovations

A key informant from NTP highlighted the need to investigate the effectiveness of a recently developed mobile application for case notification. He further emphasized the need to evaluate outcomes of introducing childhood TB guidelines in the MBBS curriculum. However, the key informants were divided on who should be engaged in undertaking research activities. Key informants from the BRAC and Damien foundation suggested that government should conduct these activities. On the other hand, government authorities reinforced that responsibility of conducting research should be undertaken by the implementing partners. However, both parties agreed that there was lack of budget for research on childhood TB.

## 4. Discussion

In this study, we explored the field and policy level facilitators and barriers to diagnosis and treatment of childhood TB in Bangladesh. Our study involved policymakers, program managers, front-line health workers, and consumers who received these services. The study identified multiple strategies to enhance the diagnosis and treatment. For example, the provision of specific guidelines for childhood TB, co-ordination between the partner agencies, capacity building of health care workers, and introduction of GeneXpert. The government’s effort in identifying childhood TB was reflected by a steady increase in case notification over the past few years [11,12,13,26]. A frequent turnover of the key managerial positions in the government health system was identified as one of the biggest challenges for implementation of the TB program in Bangladesh. As key government officials are often rotated, implementing partners find it difficult to negotiate with government for planning, design, and implementation.

Despite many efforts, Bangladesh is still lagging in case detection of childhood TB [11,12]. There is a provision for symptomatic diagnosis of TB among children younger than five years old. Doctors are required to make the clinical diagnosis. However, frequent turnover and absenteeism of pediatricians in the UHC and district hospitals contributed to delays in diagnosis and commencing treatment. It was revealed that the government has stepped up to incorporate the national TB program in MBBS curriculum. However, it will take few more years to observe the effect of this intervention as we need to wait for these medical students to graduate and start their clinical practice. Further, engaging private practitioners, such as private hospitals, paediatric clinics, and pharmacies to participate in mainstream tuberculosis control programs was identified as another key challenge. The key informants’ belief that private practitioners may be practicing a different treatment regimen is supported by a recent study in India which showed variation in TB prescription patterns among private practitioners [17]. If private practitioners can be brought under the umbrella of the national program, it can help identify more cases and ensure uniformity of treatment.

The role of community health workers is crucial in reducing the burden of a communicable disease like tuberculosis. Successful implementation of TB control programs in Nigeria and Kyrgyzstan was attributed to capacity building of health workers [34]. Extensive door to door coverage by front-line workers was key to successful immunization campaigns in Nepal [35]. Our study found that front-line workers (SS) were able to provide a clear and concise message about childhood TB. Previous studies in Bangladesh reported a lower level of knowledge on childhood TB among front-line healthcare workers [17,36]. Content-specific training modules and periodic refresher training during their monthly review meetings by their supervisors may have contributed to increased level of knowledge, attitude, and practices among the front-line workers. Although the KAP scores of the frontline workers has increased from what was previously reported in Bangladesh, their knowledge on various aspects of childhood TB was sparse despite efforts from the government and the BRAC. The BRAC and the NTP should evaluate the process and content of the refresher modules to increase the effectiveness of such refresher training.

The use of GeneXpert was linked with increased case detection of childhood TB. The GeneXpert technology can be used with sputum, blood, and other tissues such as lymph nodes. As most childhood TB cases are difficult to diagnose from the conventional sputum smear, GeneXpert may bring about a revolution in the diagnosis of childhood TB. Studies have suggested that GeneXpert may contribute to increased case detection, both in adults [37] and children [38]. The limited number of GeneXpert machines could be a potential challenge. In 2016, there were 56 GeneXpert machines across Bangladesh, mostly in large cities [12]. Key informants mentioned that number of GeneXpert machines would be increased. However, there is a probability that these machines will be concentrated in the major cities and may not be uniformly distributed to the rural areas. The government should expand the diagnostic facilities, including GeneXpert, chest X-ray, and FNAC services, to at least the Upazilla level to enhance early identification of suspected cases.

The DOTS therapy may play an important role in successful treatment. For example, in Ethiopia, DOTS improved treatment success rate from 13% in 1992 to 92% in 2013 [39]. Due to effective implementation of the DOTS program, Bangladesh has one of the highest treatment success rates for TB in the world [12]. As children frequently cough or vomit out medication, it is difficult to persuade parents to ensure their child takes medicine regularly. Repeated counselling on importance of continuing TB treatment may play an important role in TB cure. However, the challenges specific to childhood TB needs to be explored to further enhance compliance with treatment.

Due to limited IEC materials on childhood TB, it was difficult to educate parents and community. During our study, IEC materials on childhood TB were found in only two out of eight DOTS centres. The gap in availability of IEC materials was consistent with findings of a recent study from Bangladesh [17]. One of the reasons for lack of IEC materials could be a lack of accountability from the government and implementing partners. Both pointed responsibility of developing IEC materials to the other partner.

Bangladesh is globally renowned for research and innovations on tuberculosis. For example, research on a treatment regimen for multi-drug resistant tuberculosis (MDR-TB), conducted in Bangladesh, suggested reducing the treatment duration from 22 months to 9 months (the Bangladesh Regimen) [40]. However, there was no concrete roadmap for research activities on childhood TB. Lack of research activities could prevent identifying strategies improving case identification and treatment compliance.

## 5. Strength and Limitations

We conducted a mixed-methods study to review the implementation pathway of childhood TB in Bangladesh. Triangulation of findings from survey and in-depth-interviews adds to the validity of our study. Further, we had views from different stakeholders to identify key factors that enabled and hindered the implementation of childhood TB control program in Bangladesh.

There are some important limitations to consider. First, we had very limited time for data collection, analysis, and reporting of the study findings. For example, we could not manage to conduct our fieldwork beyond urban TB areas in Dhaka. A comparison between urban and rural areas and between government-operated areas and NGO (non-government organisation) operated areas could provide a better insight into the program. Second, we had to compromise on the sample size for the survey. We initially planned to determine the sample size using 90% power and 5% margin of error but that would have given us a sample size of 384 which was not possible for us with the provided time constraints. Third, we were not able to undertake some quality measures for qualitative data. We did not transcribe the interviews into Bangla before translating them to English. We were also unable to undertake translation/transcription by two researchers for all of the interviews. Not undertaking these measures could mean that we may have lost a few important participant quotes during the process of data transcription. In addition, we initially planned to conduct a focus group discussion with mothers of child TB cases, which was not feasible due to the scattered distribution of child TB cases. As a result, we conducted IDIs. We also wanted to conduct IDIs with informal health care providers, which was not possible due to time constraints. However, we believe that our study provides an important insight to the key facilitators and barriers to the implementation of childhood TB diagnosis and treatment in Bangladesh.

## 6. Conclusions

A triad of strategies including capacity building of front-line health care workers (SS), use of recent technological advances like GeneXpert, and continuous monitoring of suspected or identified cases has resulted in identification of more cases with childhood TB. We identified some important barriers to effective implementation of TB including frequent turnover of key positions in NTP, absenteeism of pediatricians in government hospitals, and underreporting of TB cases. Absence of IEC materials on child TB coupled with lack of awareness and stigma hindered childhood TB control program in Bangladesh.

## Figures and Tables

**Table 1:** Summary of the methods and respondents.

Methods		Respondents	Objectives Addressed
Quantitative Component	Survey	Shasthya Shebika * (SS) (*n* = 116)	To find out the knowledge, attitude and practices for diagnosis and treatment of childhood TB among front-line health care workers
Qualitative Component	In-depth interview (IDI) (*n* = 32)	SS (*n* = 18), Program Organizers (*n* = 6), Laboratory Technicians (*n* = 2), Area Managers (*n* = 2), Mother of children with active TB (*n* = 4)	To explore the field level challenges in diagnosis and treatment of childhood TB
	Key informant interview (KII) (*n* = 9)	BRAC (*n* = 4), National Tuberculosis Program (*n* = 2), Damien Foundation (*n* = 1), WHO National Professional Officer (*n* = 1), Tuberculosis Research Specialist (*n* = 1)	To explore the implementation pathway, strategic policies around diagnosis and treatment of childhood TB

* Frontline health worker

**Table 2:** A-priori codes for data analysis.

A-Priori Codes	Code	Sub-Code
Facilitators for Diagnosis (FD)	Human Resource	Availability of Pediatrician, doctors, health care workers, lab technicians, SS
	Lab Facilities	Chest X-ray, Mantoux test, Sputum AFB, Availability of GeneXpert
	Monetary	Financial Benefits to clients, Stipend, Incentives to service providers
Barriers for Diagnosis (BD)	Socio-economic Condition	Poverty, Education,
	Awareness	Lack of awareness, doubts about staff’s competency, belief that BCG vaccine protects children
	Human Resources	Availability of Pediatrician, doctors, health care workers, lab technicians, SS
	Lab Facilities	Chest X-ray, Mantoux test, Sputum AFB, GeneXpert
	Program Activities	Active case detection, Contact tracing, Screening Camps
	Compliance	Lack of adherence to National Guidelines
Facilitators for Treatment (FT):	Medicine	Childhood TB regimen, Child friendly drugs, Free access, Availability
	Service Delivery	Medicine delivery at home, Supervision by SS
	Monetary	Treatment bond money, Incentives to patient, Incentives to service provider
Barriers for Treatment (BT)	Socio-economic Condition	Location from DOTS center, Education, Poverty,
	Awareness	Stigma, feeling of weakness of child, belief that medicines will harm child
	Physical Conditions	Difficult in swallowing, Vomiting, Nausea, children being difficult patients, poor compliance
	Drug Properties	Taste, Size, Side effects (red urine, jaundice), lack of child friendly regimens (syrup, dispersible tablets), long treatment duration, many tablets
	Supply side	Lack of child regimen, stock out of drugs, delay in procurement

**Table 3:** Demographic Characteristics.

Category (*n* = 111 Expect Otherwise Stated)	Total Respondents *n* (%)	High Performing Centres *n* (%)	Low Performing Centres *n* (%)
Age			
<30 years	10 (9.0)	6 (11)	4 (7)
30–50 years	80 (72.1)	38 (70)	42 (74)
>50 years	21 (18.9)	10 (18)	11 (19)
Education			
Never Attended	23 (20.8)	11 (20)	12 (21)
Primary	41 (36.9)	18 (33)	23 (40)
Secondary	41 (36.9)	23 (43)	18 (32)
Higher Secondary or Above	6 (5.4)	2 (6)	4 (7)
Number of adult cases who completed treatment in past 12 months			
<2	8 (7)	2 (6)	6 (10)
“2–5”	42 (38)	23 (43)	19 (33)
“5–10”	40 (36)	18 (33)	22 (39)
> = 10	21 (19)	11 (20)	10 (18)
Duration of providing medicine to child tuberculosis cases (*n* = 40)			
<2 years	14 (35)	5 (33)	9 (36)
2–5 years	15 (38)	5 (33)	10 (40)
5–10 years	4 (10)	3 (20)	1 (4)
> = 10 years	7 (17)	2 (14)	5 (20)
Duration of Work			
<5 years	11 (10)	5 (9)	6 (10)
5–10 years	35 (31)	21 (39)	14 (25)
> = 10 years	65 (59)	28 (52)	37 (65)

**Table 4:** Facilitators and Barriers to diagnosis and treatment of childhood TB in Bangladesh.

**Name of the Pillar**	**Facilitator**	**Barrier**
Patient-Centered Care (Pillar 1)	Training and capacity building Guideline for childhood TB Child friendly regimens Financial assistance Active surveillance GeneXpert	Complexity of the disease Delay in diagnosis Lack of awareness on childhood TB
Bold Policies and Supportive System (Pillar 2)	Government ownership Separate unit for childhood TB control program Effective coordination between government and implementing organisations	Frequent turnover of high-level authorities in the ministry and NTP Gaps in regular review meetings Lack of adequate funding in childhood TB program
Intensified research ((Pillar 3)	Presence of WHO standard laboratory in Bangladesh Presence of laboratories at the regional level Engagement of some of the implementing partners in research	Lack of adequate fund for research both with NTP and with implementing partners. Lack of effective co-ordination between partners for research and innovations

## Data Availability

Additional data from the study will be made available based on institutional policy of JPGSPH.

## Supplementary Materials

The following supporting information can be downloaded at: https://www.mdpi.com/article/10.3390/nursrep12020036/s1. Supplementary material S1: Questionnaire KAP survey (English version). Supplementary material S2: STARI Checklist, and Supplementary material S3: KAP scores [31,33].
