# Supplementary material for: Facilitators and Barriers to Implementation of a Childhood Tuberculosis Control Program in Bangladesh: A Mixed-Methods Study from BRAC Urban DOTS Centres in Dhaka
Source: Nurs Rep. 2022 May 10;12(2):371–86. doi: 10.3390/nursrep12020036 (PMC9149828; doi:10.3390/nursrep12020036)
Supplement: Supplementary file 1 [file nursrep-12-00036-s001.zip › nursrep-1669600-supplementary.pdf]

nursing reports

MDPI

Supplementary File S1: Questionnaire KAP survey

Questionnaire for Shyastha Shebika for assessment of Knowledge, Awareness, and Practices on Childhood Tuberculosis

Knowledge, Awareness and Practices survey on Childhood Tuberculosis in Bangladesh

ID Number:___________ **Date of Interview:_______**

**S. No.**	**Question**	**Response**	**Code**
**Demographic Information**			
A1	Age	____ years	
A2	Name of Health Centre		
A3	Level of education	No education<br>Primary School<br>Secondary School<br>Higher Secondary<br>Undergraduate<br>Graduate	
A4	How long have you been working in the health center?	____ years	
A5	Gender	1. Male<br>2. Female	
A6	How many cases of TB did you treat/provide medicine during **last 12 months (1 year)**?	_______ (Number)	
A7	How many patients of tuberculosis that you treated in the past 1 year had been cured?	_______ (Number)	
A8	How long have you been treating/providing medicines to **children** with tuberculosis?	________ years	
A9	How many cases of **child with TB** did you provide medicine during **last 12 months (1 year)**?	_________ (Number)	
A10	How many patients of **child TB** you treated in the past 1 year had been **cured**?	_______	
A11	Which of the following children do you provide medicine of TB?		
A11.1	Under 1 year	1. Yes<br>2. No	
A11.2	1year - <5 years	1. Yes<br>2. No	
A11.3	5 years – 14 years	1. Yes<br>2. No	
**Training**			
B1	Have you ever received training on tuberculosis?	1. Yes<br>2. No	
B2.1	When was the last time you received training on TB?	______ years	
B2.2	When was the last time when you received training on TB?	_____ months (if <1 year)	
B3	Do you think the training was adequate for you to work on TB?	1. Yes<br>2. No	

		3. Do not Know
B4	Have you ever received training on childhood tuberculosis?	1. Yes<br>2. No
B5.1	When was the last time when you received training on Childhood TB?	______ years
B5.2	When was the last time when you received training on Childhood TB?	_____ months<br>(if <1 year)
B6	Have you ever been trained specifically on IPT?	1. Yes<br>2. No
B7	If yes what was the duration of IPT training?	_____ days
B8	Was that training on (IPT) childhood TB adequate for your work?	1. Yes<br>2. No<br>3. Do not Know
B9	How often are you getting refresher training?	1. every month<br>2. Once in 3 months<br>3. Once in 6 months<br>4. Once in a year
B10	What is the duration of the refresher training?	________ day
B11	Do you think refresher training is adequate imparting knowledge and skills?	1. Yes<br>2. No
B12	What components are being covered during training?	
B12.1	Diagnosis	1. Yes<br>2. No<br>3. Do not Know
B12.2	Treatment	1. Yes<br>2. No<br>3. Do not Know
B12.3	Prevention	1. Yes<br>2. No<br>3. Do not Know
B12.4	IPT	1. Yes<br>2. No<br>3. Do not Know
B12.5	Counseling	1. Yes<br>2. No<br>3. Do not Know
B12.6	Stigma	1. Yes<br>2. No<br>3. Do not Know
B12.7	Management/maintaining Stock and Supply of medicine/sputum pot/mask	1. Yes<br>2. No<br>3. Do not Know
**Knowledge on Tuberculosis**		
C1	How many sputum samples are necessary for diagnosis?	________ (Number)
C2	How should a sputum sample be stored before laboratory analysis?	1. At room temperature

		2. Sputum samples cannot be stored – should be sent to the laboratory immediately<br>3. In the fridge<br>4. In culture medium
C3	Do you think we need to do sputum examination after starting treatment? (if yes go to # , else go to #)	1. Yes<br>2. No<br>3. Do not Know
C4	After initiation of the treatment, when the next sputum examination should be done?	1. after 1 month<br>2. after 2 months<br>3. after 3 months<br>4. after 5 month
C5	If the sputum is positive at the end of 2 months, when should the next examination be repeated?	1. Repeat the next day<br>2. Repeat after 1 week<br>3. Repeat after 1 month<br>4. At the end of 5 months of treatment
C6	Is tuberculosis a transmissible disease?	1. Yes<br>2. No
C7	Does tuberculosis spread from the following routes (Knowledge on route of transmission)	
C7.1	By coughing/sneezing	1. Yes<br>2. No<br>3. Do not Know
C7.2	By direct contact	1. Yes<br>2. No<br>3. Do not Know
C7.3	By sharing room	1. Yes<br>2. No<br>3. Do not Know
C7.4	By sharing food (eating from same plate)	1. Yes<br>2. No<br>3. Do not Know
C7.5	By sharing utensils/towels	1. Yes<br>2. No<br>3. Do not Know
C7.6	From Sexual Intercourse	1. Yes<br>2. No<br>3. Do not Know
C7.7	By sharing needles	1. Yes<br>2. No<br>3. Do not Know
C7.8	During pregnancy	1. Yes

		2. No<br>3. Do not Know
C7.9	During Childbirth	1. Yes<br>2. No<br>3. Do not Know
C7.10	Through breastfeeding	1. Yes<br>2. No<br>3. Do not Know
C8	How can we prevent spread of TB? (Knowledge on route of transmission)	
C8.1	Covering mouth and nose while coughing or sneezing	1. Yes<br>2. No<br>3. Do not Know
C8.2	Avoid sharing dishes	1. Yes<br>2. No<br>3. Do not Know
C8.3	Washing hands	1. Yes<br>2. No<br>3. Do not Know
C8.4	Closing windows at home	1. Yes<br>2. No<br>3. Do not Know
C8.5	Good nutritious foods	1. Yes<br>2. No<br>3. Do not Know
C8.6	Praying	1. Yes<br>2. No<br>3. Do not Know
**Knowledge on Childhood TB**		
D1	Do you think **children** under 5 years can have tuberculosis?	1. Yes<br>2. No<br>3. Do not Know
D2	Do you think **children** between 5 years to 14 years can have tuberculosis?	1. Yes<br>2. No<br>3. Do not Know
D3	Can any vaccine protect children against tuberculosis?	1. Yes<br>2. No<br>3. Do not Know
D4	If yes, which vaccine can protect children against tuberculosis?	1. DPT<br>2. Measles<br>3. BCG<br>4. Polio
D5	Do you think BCG vaccine protect **children** against Tuberculosis?	1. Yes<br>2. No<br>3. Do not Know
D6	Do you think BCG vaccine protect **all children** against Tuberculosis?	1. Yes<br>2. No

		3. Do not Know
D7	When is the best time for BCG vaccination?	1. At birth<br>2. At 6 weeks<br>3. At 10 months<br>4. At 14 weeks<br>5. Other. Specify__________
D8	Is TB diagnosis in children more difficult than in adults?	1. Yes<br>2. No
D9	Is a child with following conditions at higher risk of developing tuberculosis?	
D9.1	Malnourished Child	1. Yes<br>2. No<br>3. Do not Know
D9.2	Child with any form of TB in family	1. Yes<br>2. No<br>3. Do not Know
D9.3	Child with sputum positive TB in family	1. Yes<br>2. No<br>3. Do not Know
D9.4	Child with measles in past 3 months	1. Yes<br>2. No<br>3. Do not Know
D9.5	Child with recent weight loss	1. Yes<br>2. No<br>3. Do not Know
**Knowledge on Symptoms of Childhood TB**		
D10	Are the following symptoms of Tuberculosis in children	
D10.1	Fever of >2wk	1. Yes<br>2. No<br>3. Do not Know
D10.2	Loss of appetite	1. Yes<br>2. No<br>3. Do not Know
D10.3	Failure to gain weight	1. Yes<br>2. No<br>3. Do not Know
D10.4	Loss of weight	1. Yes<br>2. No<br>3. Do not Know
D10.5	Persistent cough (>3 weeks)	1. Yes<br>2. No<br>3. Do not Know
D10.6	Lethargy (less playful child)	1. Yes<br>2. No<br>3. Do not Know
D10.7	Abdominal Pain	1. Yes

		2. No<br>3. Do not Know
D10.8	Body ache	1. Yes<br>2. No<br>3. Do not Know
D10.9	Coughing with blood	1. Yes<br>2. No<br>3. Do not Know
D10.10	Weakness	1. Yes<br>2. No<br>3. Do not Know
D10.11	Throat Pain	1. Yes<br>2. No<br>3. Do not Know
D10.12	Mass/ Swelling in the neck	1. Yes<br>2. No<br>3. Do not Know
D10.13	Mass/Swelling in the back	1. Yes<br>2. No<br>3. Do not Know
**Knowledge on Treatment of Tuberculosis**		
E1	Is tuberculosis in children a curable disease?	1. Yes<br>2. No<br>3. Do not Know
E2	Is there a difference in duration for treatment of TB in child and in adult?	1. Yes<br>2. No<br>3. Do not Know
E3	How many phases are there in the treatment of TB?	____________
E4	What is the duration of treatment of pulmonary (lungs) tuberculosis in children?	2 months<br>6 months<br>9 months<br>12 months
E5	How long is the intensive phase (first phase) of pulmonary (lungs) tuberculosis in children?	1 month<br>2 months<br>3 months<br>4 months
E6	How many types of drugs are used in the first two months of treatment of tuberculosis (in the intensive phase)?	__________
E7	How many types of drugs are used in the remaining period of treatment of tuberculosis (in the continuation phase)?	__________
E8	Can adult TB drugs be provided to the children?	1. Yes<br>2. No<br>3. Do not Know
E9	What are the consequences of incomplete TB treatment (in children)?	
E9.1	Disease may not be cured	1. Yes<br>2. No

		3. Do not Know
E9.2	Disease will worsen	1. Yes<br>2. No<br>3. Do not Know
E9.3	Further transmission of the disease	1. Yes<br>2. No<br>3. Do not Know
E9.4	Relapse of the disease	1. Yes<br>2. No<br>3. Do not Know
E9.5	Child may Develop Resistant TB	1. Yes<br>2. No<br>3. Do not Know
E9.6	Child may die	1. Yes<br>2. No<br>3. Do not Know
**Attitudes on Diagnosis and Treatment**		
F1	Finding every new case of tuberculosis is essential for control of the disease	1. Yes<br>2. No<br>3. Do not Know
F2	Community engagement is essential for the control of the disease	1. Yes<br>2. No<br>3. Do not Know
F3	There is a substantial increase in treatment completion rates if direct observed treatment (DOTS) is used	1. Yes<br>2. No<br>3. Do not Know
F4	Making people with suspected/confirmed pulmonary tuberculosis wear masks at home is acceptable	1. Yes<br>2. No<br>3. Do not Know
F5	Teaching cough hygiene to tuberculosis patients is not important	1. Yes<br>2. No<br>3. Do not Know
F6	Public awareness regarding childhood tuberculosis is not sufficient in your community	1. Yes<br>2. No<br>3. Do not Know
F7	In your community are people aware of services that are available for children with TB	1. Yes<br>2. No<br>3. Do not Know
F8	How can TB in a child be cured?	
F8.1	Herbal Medicines	1. Yes<br>2. No<br>3. Do not Know
F8.2	Home without specific Treatment	1. Yes<br>2. No<br>3. Do not Know
F8.3	Praying	1. Yes

		2. No<br>3. Do not Know
F8.4	Drugs given by informal care provider	1. Yes<br>2. No<br>3. Do not Know
F8.5	DOTS	1. Yes<br>2. No<br>3. Do not Know
**Practices on Diagnosis and Treatment:**		
G1	How frequently do you provide medicines to a child with TB?	Daily<br>Weekly<br>Once in 2 weeks<br>Monthly
G2	How are the drugs supplied?	Patients come to my home<br>I go to patient's house home<br>Patients take drug from the DOTS center
G3	How do the patients take the drugs?	In front of me<br>In front of the father of the child<br>In front of the mother of the child<br>Do not know; I just provide them medicine
G4	Have you ever provided medicine to a child by splitting/crushing an adult tablet?	Yes, often<br>Yes, but occasionally<br>No
G5	Do you counsel a TB patient on the following aspects of TB during diagnosis and treatment?	
G5.1	TB is a curable disease	1. Yes<br>2. No<br>3. Do not Know
G5.2	Duration of treatment	1. Yes<br>2. No<br>3. Do not Know
G5.3	Importance of compliance	1. Yes<br>2. No<br>3. Do not Know
G5.4	Importance of follow up	1. Yes<br>2. No<br>3. Do not Know
G5.5	Side effects	1. Yes<br>2. No<br>3. Do not Know
G5.6	Prevention of spread of disease	1. Yes

		2. No<br>3. Do not Know
G6	What do you think can prevent patients from missing their treatment?	
G6.1	Set up feeding program for medicines	1. Yes<br>2. No
G6.2	Intensifying Health education	1. Yes<br>2. No
G6.3	Admission at health facility	1. Yes<br>2. No
G6.4	Give all medicine to take home	1. Yes<br>2. No
G6.5	Take medicine from you every day	1. Yes<br>2. No
G7	How often do you measure the weight of a child under-5 years of age during treatment?	1. Once in a week<br>2. Once in 2 weeks<br>3. Once in a month<br>4. Once in 2 months
G8	Do you think is it important to measure the weight during treatment?	1. Yes<br>2. No
G9	Do you have a National guideline on management of Childhood TB?	1. Yes<br>2. No
**Knowledge about IPT**		
H1	In your opinion, do you think that IPT reduces the risk of TB infection in children?	1. Yes<br>2. No
H2	Do you think IPT reduces the risk of Tuberculosis in children for a period?	1. Yes<br>2. No
H3	In your opinion, who is eligible to receive IPT?	
H3.1	All children < 5 yrs of parents with pulmonary TB	1. Yes<br>2. No
H3.2	All children < 5 yrs in contact with smear positive TB patient	1. Yes<br>2. No
H3.3	All Children (5–14) years of parent with pulmonary TB	1. Yes<br>2. No
H3.4	All children (5–14) yrs in contact with smear positive TB patient	1. Yes<br>2. No
**Attitudes on IPT**		
I1	What is the duration of protective effect of one cycle of IPT?	________
I2	Is it important to take IPT medicine regularly?	1. Yes<br>2. No
I3	How do you ensure whether your patients are taking IPT medicines regularly or not?	1. Self-report from caretaker<br>2. Detailed interview with caretaker<br>3. Pill counts<br>4. Pharmacy refill data

		5. All above<br>6. None of the above
I4	IPT is helpful in reducing TB burden among Children.	1. Yes<br>2. No<br>3. Do not Know
I5	Can children with any TB symptoms (current cough, fever, weight loss or night sweats) can be offered IPT.	1. Yes<br>2. No<br>3. Do not Know
I6	IPT should be given to all eligible children irrespective of their age.	1. Yes<br>2. No<br>3. Do not Know
I7	Children < 1 year of age should be provided with IPT if they have a household contact history with a TB case	1. Yes<br>2. No<br>3. Do not Know
I8	Children 1- < 5years of age should be provided with IPT if they have a household contact history with a TB case	1. Yes<br>2. No<br>3. Do not Know
I9	Children 5–14 year of age should be provided with IPT if they have a household contact history with a TB case.	1. Yes<br>2. No<br>3. Do not Know
I10	History of TB (>2 years) is not a contraindication for IPT provision	1. Yes<br>2. No<br>3. Do not Know
I11	Do you use any TB screening guideline to identify children eligible for IPT?	1. Yes, always<br>2. Yes, sometimes<br>3. No
I12	Do you encourage parents of the children to start IPT once they are eligible?	1. Yes, always<br>2. Yes, sometimes<br>3. No
I13	Do you provide IPT for eligible child patients? (If no go to Q I17)	1. Yes, always<br>2. Yes, sometimes<br>3. No (
I14	If your answer for Q13 is 'Yes', how many of them started IPT in the last one year?	____
I15	If you are providing IPT for your patients, how frequent do you supply the drugs?	1. Daily<br>2. Weekly<br>3. Once in 2 weeks<br>4. Once in a month
I16	Do you advise clients to take medicines (IPT) regularly?	1. Yes, always<br>2. Yes, sometimes<br>3. No
I17	Do you monitor and manage clients with IPT side effects?	1. Yes, always<br>2. Yes, sometimes<br>3. No
I18		1. Yes

	Is it necessary to provide IPT to children every time they are exposed to a TB patient at home/close contact?	2. No<br>3. Do not Know
**Knowledge on Human Resources Management**		
J1	Are you being regularly supervised by your immediate supervisor/manager for your works?	1. Yes<br>2. No
J2	How often the supervision happens?	Monthly<br>Once in 3 months<br>Once in 6 months<br>Once in a year
J3	When was the last time you have been supervised by your supervisor?	Last week<br>2 weeks ago<br>3 weeks ago<br>Last month
J4	Did your last supervision visit include drug management (patient card checked; drug card checked etc.)?	1. Yes<br>2. No
J5	Does your immediate supervisor/manager provide feedback on your works/reports?	1. Yes<br>2. No
**Attitudes on Human Resource Management**		
K1	The contents of the training programs organized are always relevant to the changing needs of our job	1. Yes<br>2. No<br>3. Do not Know
K2	I am being paid adequately for the work I do	1. Yes<br>2. No<br>3. Do not Know
K3	Incentive plans do not motivate me for better performance.	1. Yes<br>2. No<br>3. Do not Know
K4	I receive proper feedback on how I am performing	1. Yes<br>2. No<br>3. Do not Know
K5	My supervisor encourages me to discuss my problems with him/her	1. Yes<br>2. No<br>3. Do not Know
K6	Adequate training regarding childhood TB facilitates TB management	1. Yes<br>2. No<br>3. Do not Know
K7	Regular supervision/proper guidance helps to improve performance	1. Yes<br>2. No<br>3. Do not Know
K8	Availability of required facilities (drugs, sputum cup, face mask etc) improves quality of work	1. Yes<br>2. No<br>3. Do not Know
K9	Good working relations with supervisor helps to improve performance	1. Yes<br>2. No<br>3. Do not Know
K10	Financial incentives motivate me to work	1. Yes

		2.	No
		3.	Do not Know
K11	Frequency of refresher training is adequate	1.	Yes
		2.	No
		3.	Do not Know
K12	Enough IEC materials for health education are available	1.	Yes
		2.	No
		3.	Do not Know
K13	Frequency of supervision is adequate	1.	Yes
		2.	No
		3.	Do not Know
K14	I have got required communications skills to talk about the TB in children for public?	1.	Yes
		2.	No
		3.	Do not Know
K15	I have got skills to collect sputum from children with suspected TB	1.	Yes
		2.	No
		3.	Do not Know
K16	Do you have Handbooks on TB in children for health education?	1.	Yes
		2.	No
K17	Do you have any IEC materials (posters, pamphlets, flipchart, leaflets etc.) on TB in children for health education?	1.	Yes
		2.	No
K18	Do you have regular monthly meeting with supervisor?	1.	Yes
		2.	No
**Supply Chain Management**			
L1	In the past 3 months did you faced any interruption in DOTS services?	1.	Yes
		2.	No
L2	In the past 3 months did you faced any interruption in supply of anti-TB drugs?	1.	Yes
		2.	No
L3	In the past 3 months did you faced any interruption in supply of pediatric/childhood anti-TB drugs?	1.	Yes
		2.	No
L4	In the past 3 months did you faced any interruption in supply of infection control equipments (gloves, aprons, gowns, caps, masks etc.)?	1.	Yes
		2.	No
L5	In the past 3 months did you faced any interruption in supply of sputum collection pot?	1.	Yes
		2.	No
L6	Do you have any IEC materials on TB in children for health education sufficient for 1 month?	1.	Yes
		2.	No
L7	Do you know how to complete the patient card/patient information sheet/drug card?	1.	Yes
		2.	No
L8	How did you learn to complete the patient card/patient information sheet/drug card?	During a training on tuberculosis	
		On-the-job training from supervisor	
		Informal learning from other colleagues	
		Self-learning	

		Never been trained
L9	How many emergency orders for DOTS service (e.g. anti-TB drugs, infection control accessories etc.) have you placed in the last 3 months?	None<br>1<br>2<br>3<br>More than 3
L10	How many emergency orders for childhood anti-TB drugs for DOTS service have you placed in the last 3 months?	None<br>1<br>2<br>3<br>More than 3
L11	How many emergency orders of supplies (sputum collection pots, masks etc.) have you placed in the last 3 months?	None<br>1<br>2<br>3<br>More than 3
L12	How do you calculate the quantities of anti-TB drugs?	Number of TB cases<br>Any formula<br>Don't know
L13	How do you get the products (anti-TB drugs, sputum collection pot, mask etc.)?	Program officer delivers<br>Field organizer delivers<br>I collect myself<br>Other ___________
L14	On average, approximately how long does it take between ordering and receiving products?	On the same day<br>Less than one week<br>Less than two weeks<br>Less than one month<br>More than one month<br>More than two month

Supplementary File S2: Standards for Reporting Implementation Studies: the StaRI checklist for completion

StaRI
Standards for reporting implementation studies

The StaRI standard should be referenced as:

Pinnock H, Barwick M, Carpenter C, Eldridge S, Grandes G, Griffiths CJ, Rycroft-Malone J, Meissner P, Murray E, Patel A, Sheikh A, Taylor SJC for the StaRI Group. Standards for Reporting Implementation Studies (StaRI) statement. *BMJ* 2017;356:i6795

The detailed Explanation and Elaboration document, which provides the rationale and exemplar text for all these items is:

Pinnock H, Barwick M, Carpenter C, Eldridge S, Grandes G, Griffiths C, Rycroft-Malone J, Meissner P, Murray E, Patel A, Sheikh A, Taylor S, for the StaRI group. Standards for Reporting Implementation Studies (StaRI). Explanation and Elaboration document. *BMJ Open* 2017 2017;7:e013318

Notes: A key concept of the StaRI standards is the dual strands of describing, on the one hand, the implementation strategy and, on the other, the clinical, healthcare, or public health intervention that is being implemented. These strands are represented as two columns in the checklist.

The primary focus of implementation science is the implementation strategy (column 1) and the expectation is that this will always be completed.	The evidence about the impact of the intervention on the targeted population should always be considered (column 2) and either health outcomes reported or robust evidence cited to support a known beneficial effect of the intervention on the health of individuals or populations.

The StaRI standards refers to the broad range of study designs employed in implementation science. Authors should refer to other reporting standards for advice on reporting specific methodological features. Conversely, whilst all items are worthy of consideration, not all items will be applicable to, or feasible within every study.

Checklist item		Reported on page #	Implementation Strategy	Reported on page #	Intervention
			"Implementation strategy" refers to how the intervention was implemented		"Intervention" refers to the healthcare or public health intervention that is being implemented.
**Title and abstract**					
Title	**1**		Identification as an implementation study, and description of the methodology in the title and/or keywords		

		2			
Abstract	**2**	2–3	Identification as an implementation study, including a description of the implementation strategy to be tested, the evidence-based intervention being implemented, and defining the key implementation and health outcomes.		
**Introduction**					
Introduction	**3**	2–3	Description of the problem, challenge or deficiency in healthcare or public health that the intervention being implemented aims to address.		
Rationale	**4**	2–4	The scientific background and rationale for the implementation strategy (including any underpinning theory/framework/model, how it is expected to achieve its effects and any pilot work).		The scientific background and rationale for the intervention being implemented (including evidence about its effectiveness and how it is expected to achieve its effects).
Aims and objectives	**5**	2–3	The aims of the study, differentiating between implementation objectives and any intervention objectives.		
**Methods: description**					
Design	**6**	3–4	The design and key features of the evaluation, (cross referencing to any appropriate methodology reporting standards) and any changes to study protocol, with reasons		
Context	**7**	2–4	The context in which the intervention was implemented. (Consider social, economic, policy, healthcare, organisational barriers and facilitators that might influence implementation elsewhere).		
Targeted 'sites'	**8**	3–4	The characteristics of the targeted 'site(s)' (e.g locations/personnel/resources etc.) for implementation and any eligibility criteria.	3–4	The population targeted by the intervention and any eligibility criteria.
Description	**9**	5–6	A description of the implementation strategy	5–6	A description of the intervention
Sub-groups	**10**	N/A	Any sub-groups recruited for additional research tasks, and/or nested studies are described		
**Methods: evaluation**					

Outcomes	**11**	N/A	Defined pre-specified primary and other outcome(s) of the implementation strategy, and how they were assessed. Document any pre-determined targets	N/A	Defined pre-specified primary and other outcome(s) of the intervention (if assessed), and how they were assessed. Document any pre-determined targets
Process evaluation	**12**	5–8	Process evaluation objectives and outcomes related to the mechanism by which the strategy is expected to work		
Economic evaluation	**13**	N/A	Methods for resource use, costs, economic outcomes and analysis for the implementation strategy	N/A	Methods for resource use, costs, economic outcomes and analysis for the intervention
Sample size	**14**	3–4	Rationale for sample sizes (including sample size calculations, budgetary constraints, practical considerations, data saturation, as appropriate)		
Analysis	**15**	4–5	Methods of analysis (with reasons for that choice)		
Sub-group analyses	**16**	N/A	Any a priori sub-group analyses (e.g. between different sites in a multicentre study, different clinical or demographic populations), and sub-groups recruited to specific nested research tasks		
**Results**					
Characteristics	**17**	5–6	Proportion recruited and characteristics of the recipient population for the implementation strategy		Proportion recruited and characteristics (if appropriate) of the recipient population for the intervention
Outcomes	**18**	6–10	Primary and other outcome(s) of the implementation strategy		Primary and other outcome(s) of the Intervention (if assessed)
Process outcomes	**19**	N/A	Process data related to the implementation strategy mapped to the mechanism by which the strategy is expected to work		
Economic evaluation	**20**	N/A	Resource use, costs, economic outcomes and analysis for the implementation strategy		Resource use, costs, economic outcomes and analysis for the intervention
Sub-group analyses	**21**	N/A	Representativeness and outcomes of subgroups including those recruited to specific research tasks		

Fidelity/ adaptation	**22**	N/A	Fidelity to implementation strategy as planned and adaptation to suit context and preferences		Fidelity to delivering the core components of intervention (where measured)
Contextual changes	**23**	N/A	Contextual changes (if any) which may have affected outcomes		
Harms	**24**	N/A	All important harms or unintended effects in each group		
**Discussion**					
Structured discussion	**25**	7–10	Summary of findings, strengths and limitations, comparisons with other studies, conclusions and implications		
Implications	**26**	11	Discussion of policy, practice and/or research implications of the implementation strategy (specifically including scalability)	11	Discussion of policy, practice and/or research implications of the intervention (specifically including sustainability)
**General**					
Statements	**27**	12	Include statement(s) on regulatory approvals (including, as appropriate, ethical approval, confidential use of routine data, governance approval), trial/study registration (availability of protocol), funding and conflicts of interest		

Adapted from Pinnock et al. (2017):

Pinnock, H.; Barwick, M.; Carpenter, C.; Eldridge, S.; Grandes, G.; Griffiths, C.J.; Rycroft-Malone, J.; Meissner, P.; Murray, E.; Patel, A.; et al. Standards for Reporting Implementation Studies (StaRI) Statement. *BMJ* **2017**, *356*, i6795. https://doi.org/10.1136/bmj.i6795.

Supplementary File S3: KAP scores

Mean (± S.E.) and 95% CI of scores on Knowledge, Attitude and Practice of childhood TB

Traits	Mean (± S.E.)	95% CI
**Total Knowledge Score (*n* = 61)**	**43.6 (± 0.5)**	**42.6–44.6**
1. Knowledge on Tuberculosis (n = 22)	12 (± 0.2)	11.6–12.40
Knowledge on Transmission of Disease (*n* = 10)	4.3 (± 0.3)	4.0 -4.6
Knowledge on Prevention of Spread of disease (*n* = 6)	4.0 (± 0.1)	3.8–4.2
2. Knowledge on Child TB (n = 27)	22.0 (± 0.3)	21.4–22.6
Knowledge Risk factor for Child TB (*n* = 5)	4.3 (± 0.1)	4.2–4.5
Knowledge Symptoms of Child Tuberculosis (*n* = 13)	11.1 (± 0.2)	10.7–11.5
3. Knowledge on Treatment of Tuberculosis (n = 12)	9.5 (± 0.1)	9.3–9.7
Technical knowledge (*n* = 6)	3.7 (± 0.2)	3.3–4.1
Knowledge Consequence of Incomplete treatment (*n* = 6)	5.8 (± 0.1)	5.6–6.0
**Attitude on Child Tuberculosis (*n* = 12)**	**10.8 (± 0.9)**	**9–11.6**
Attitude on Ways to cure (*n* = 5)	4.8 (± 0.05)	4.7–4.9
**Practice of Diagnosis and Treatment (*n* = 16)**	**13.2 (± 0.2)**	**12.8–13.6**
Practice on Drug Dispensing (*n* = 5)		
Practice on Counseling (*n* = 6)	5.7 (± 0.1)	5.5–5.9
Practice on ensuring compliance (*n* = 5)	3.9 (± 0.1)	3.7–4.1

Frequency and percentage distribution of Knowledge, Attitude, and Practice Scores on childhood TB

Trait	Poor Score *n* (percentage)	Average Score *n* (percentage)	Good Score *n* (percentage)
Knowledge on Sputum processing	7 (6.3)	46 (41.4)	58 (52.3)
Knowledge on Transmission of TB	33 (29.7)	73 (65.8)	5 (4.5)
Knowledge on Prevention of Spread of TB	5 (4.5)	68 (61.3)	38 (34.2)
Knowledge Risk factor for Child TB	5 (4.5)	9 (8.1)	97 (97.4)
Knowledge Symptom Child TB	0 (0)	25 (22.5)	86 (77.5)
Overall Knowledge on Child TB	1 (0.9)	12 (10.8)	98 (88.3)
Knowledge on technical aspects of treatment	23 (20.7)	54 (48.7)	34 (30.6)
Consequence of Incomplete treatment	0 (0)	3 (2.7)	108 (97.3)
Knowledge on Treatment of TB	0 (0)	28 (25.2)	83 (74.8)
**Total Knowledge Score**	**0 (0)**	**36 (32.4)**	**75 (67.6)**
**Attitude on Child TB**	**0 (0)**	**1 (0.9)**	**110 (99.1)**
Practice on Drug Dispensing	5 (4.5)	11 (9.1)	95 (85.4)
Practice on Counseling	4 (3.6)	0 (0)	107 (96.4)
Practice on ensuring compliance	4 (3.6)	24 (21.6)	83 (74.8)
**Practice of Diagnosis and Treatment**	**4 (3.6)**	**3 (2.7)**	**104 (93.7)**

Knowledge of Tuberculosis among the Shasthya Shebika

Traits	Average Knowledge	Good Knowledge	*p*-value
**Age (*n* = 111)**			0.519*
< 30 years	10	0	
30–50 years	68	12	
>50 years	19	2	
**Education (*n* = 111)**			0.76
Never Attended	21	2	
Primary	36	5	
Secondary or above	40	7	
**Work Duration (*n* = 111)**			0.202*
<5 years	11	0	
5–10 years	32	3	
10 years or more	54	11	
**Number of tuberculosis patient treated in last 1 year (*n* = 110)**			0.15
less than 10	64	12	
10 or more	32	2	
**Number of tuberculosis patient cured in last 1 year (*n* = 108)**			0.213
less than 10	74	13	
10 or more	20	1	
**Performance of the DOTS center (*n* = 111)**			0.914
High Performing Center	47	7	
Low performing Center	50	7	
**Components of training (*n* = 111)**			0.636
less than 5	11	1	
more than 5	86	13	

Knowledge Childhood TB

Dependent Variables	Poor Knowledge	Average Knowledge	Good Knowledge	*p*-value*
**Age (*n* = 111)**				0.137
< 30 years	1	1	8	
30–50 years	0	10	70	
>50 years	0	1	20	
**Education (*n* = 111)**				0.505
Never Attended	0	1	22	
Primary	0	4	37	
Secondary or above	1	7	39	
**Work Duration (*n* = 111)**				0.887
<5 years	0	1	10	
5–10 years	0	5	30	
10 years or more	1	6	58	
**Number of tuberculosis patient treated in last 1 year (*n* = 110)**				0.54
less than 10	1	10	65	
10 or more	0	2	32	
**Number of tuberculosis patient cured in last 1 year (*n* = 108)**				0.56
less than 10	1	11	75	
10 or more	0	1	20	
**Performance of the DOTS center (*n* = 111)**				0.55
High Performing Center	0	7	47	
Low performing Center	1	5	51	
**Components of training (*n* = 111)**				0.219
less than 5	0	3	9	
more than 5	1	9	89	

* Fischer exact

Knowledge on Symptoms Child TB

Traits	Average Knowledge	Good Knowledge	*p*-value
**Age (*n* = 111)**			0.249*
< 30 years	3	7	
30–50 years	20	60	
>50 years	2	19	
**Education (*n* = 111)**			0.191
Never Attended	2	21	
Primary	10	31	
Secondary or above	13	34	
**Work Duration (*n* = 111)**			0.936
<5 years	2	9	
5–10 years	8	27	
10 years or more	15	50	
**Number of tuberculosis patient treated in last 1 year (*n* = 110)**			***0.02***
less than 10	22	54	
10 or more	3	31	
**Number of tuberculosis patient cured in last 1 year (*n* = 108)**			***0.09***
less than 10	23	64	
10 or more	2	19	
**Performance of the DOTS center (*n* = 111)**			0.703
High Performing Center	13	41	
Low performing Center	12	45	
**Components of training (*n* = 111)**			0.342
less than 5	4	8	
more than 5	21	78	
**Duration of treating child tuberculosis patient (*n* = 40)**			0.175*
<5 years	11	14	
5–10 years	0	4	
10 years or more	2	9	

* Fischer exact

Total Knowledge Score

Traits	Average Knowledge	Good Knowledge	*p*-value
**Age (*n* = 111)**			0.449
< 30 years	5	5	
30–50 years	25	55	
>50 years	6	15	
**Education (*n* = 111)**			0.964
Never Attended	8	15	
Primary	13	28	
Secondary or above	15	32	
**Work Duration (*n* = 111)**			0.898
<5 years	4	7	
5–10 years	12	23	
10 years or more	20	45	
**Number of tuberculosis patient treated in last 1 year (*n* = 110)**			0.212
less than 10	27	49	
10 or more	8	26	
**Number of tuberculosis patient cured in last 1 year (*n* = 108)**			0.399
less than 10	29	58	
10 or more	5	16	
**Performance of the DOTS center (*n* = 111)**			0.546
High Performing Center	19	35	
Low performing Center	17	40	
**Components of training (*n* = 111)**			***0.042***
less than 5	7	5	
more than 5	29	70	
**Duration of treating child tuberculosis patient (*n* = 40)**			0.506*
<5 years	7	18	
5–10 years	0	4	
10 years or more	4	7	

* Fischer Exact test

Attitude on Diagnosis and Treatment of Child Tuberculosis

Traits	Aver-age	Good	*p*-value*
**Age (*n* = 111)**			0.822
< 30 years	0	10	
30–50 years	1	79	
>50 years	0	21	
**Education (*n* = 111)**			1
Never Attended	0	23	
Primary	0	41	
Secondary or above	1	46	
**Work Duration (*n* = 111)**			0.414
<5 years	0	11	
5–10 years	1	34	
10 years or more	0	65	
**Number of tuberculosis patient treated in last 1 year (*n* = 110)**			
less than 10	0	76	
10 or more	1	33	
**Number of tuberculosis patient cured in last 1 year (*n* = 108)**			0.806
less than 10	1	86	
10 or more	0	21	
**Duration of treatment of Child TB (*n* = 40)**			1
<5 years	1	24	
5–10 years	0	4	
10 years or more	0	11	
**Performance of the DOTS center (*n* = 111)**			0.328
High Performing Center	0	54	
Low performing Center	1	56	
**Components of training (*n* = 111)**			0.108
less than 5	1	11	
more than 5	0	99	

* Fischer exact

Practice on Diagnosis and Treatment of Child Tuberculosis

Traits	Poor	Average	Good	*p*-value*
**Age (*n* = 111)**				0.585
< 30 years	1	0	9	
30–50 years	2	3	75	
>50 years	1	0	20	
**Work Duration (*n* = 111)**				0.634
<5 years	1	0	22	
5–10 years	2	0	39	
10 years or more	1	3	43	
**Number of tuberculosis patient treated in last 1 year (*n* = 110)**				1
less than 10	3	2	71	
10 or more	1	1	32	
**Number of tuberculosis patient cured in last 1 year (*n* = 108)**				0.736
less than 10	3	2	82	
10 or more	0	1	20	
**Duration of treatment of Child TB (*n* = 40)**				***0.064***
<5 years	0	0	25	
5–10 years	0	1	3	
10 years or more	1	0	10	
**Performance of the DOTS center (*n* = 111)**				0.144
High Performing Center	0	2	52	
Low performing Center	4	1	52	
**Components of training (*n* = 111)**				0.562
less than 5	1	0	11	
more than 5	3	3	93	

* Fischer exact
